# Smart Irrigation with Fuzzy Decision Support Systems in Trentino Vineyards

**DOI:** 10.3390/s25237188

**Published:** 2025-11-25

**Authors:** Romeo Silvestri, Massimo Vecchio, Miguel Pincheira, Fabio Antonelli

**Affiliations:** Fondazione Bruno Kessler, 38122 Trento, Italy; rsilvestri@fbk.eu (R.S.); mpincheiracaro@fbk.eu (M.P.); fantonelli@fbk.eu (F.A.)

**Keywords:** IoT-based agriculture, irrigation decision support systems, precision agriculture, soil moisture forecasting, sustainable water management

## Abstract

Efficient water management is a critical challenge for modern agriculture, particularly in the context of increasing climate variability and limited freshwater resources. This study presents a comparative field-based evaluation of two fuzzy-logic-based irrigation decision support systems for vineyard management: a Mamdani-type controller with expert-defined rules and a Takagi–Sugeno system designed to enable automated learning from ultra-local historical field data. Both systems integrate soil moisture sensing, short-term forecasting, and weather predictions to provide optimized irrigation recommendations. The evaluation combines counterfactual simulations with a bootstrap-based statistical analysis to assess water use efficiency, soil moisture control, and robustness to environmental variability. The comparison highlights distinct strengths of the two approaches, revealing trade-offs between water conservation and crop stress mitigation, and offering practical insights for the design and deployment of intelligent irrigation management solutions.

## 1. Introduction

Water scarcity is rapidly emerging as one of the most pressing global challenges of the 21st century. As emphasized by the United Nations Sustainable Development Goals, particularly SDG 6 on clean water and sanitation, the sustainable management of freshwater resources is essential for human well-being, food security, and environmental resilience [1]. Although in some regions water is still perceived as abundant and readily available, this perception is increasingly outdated due to the impacts of climate change, population growth, and expanding agricultural demand. Agriculture alone accounts for over 70% of global freshwater withdrawals, making irrigation a critical area for strategic intervention [2].

Recent advances in remote sensing, variable-rate application systems, autonomous machinery, and Internet of Things (IoT) networks are generating unprecedented volumes of field data [3,4]. When combined with AI-driven analytics, these technologies can support real-time monitoring and adaptive control, reducing water consumption while sustaining yields [5]. Unlike in fully autonomous Industry 4.0 settings, many precision irrigation scenarios can operate with low-cost sensors and actuators, offloading computationally intensive tasks to intermediate gateways or the cloud [6]. Such architectures lower field-level costs while enabling advanced processing and decision-support capabilities. Despite this potential, irrigation management faces persistent technical and economic barriers: models must remain accurate across heterogeneous microclimates, recommendations must be interpretable to build farmer trust [7], and solutions must be viable within the narrow profit margins of farming. Local environmental variability—even within a single field—complicates decision-making, and the growing frequency of extreme weather events demands systems that adapt rapidly to changing conditions. These needs point toward hybrid decision-making approaches that combine ultra-local sensing, which in this work refers to very high-resolution measurements collected directly at the sensor location (i.e., a soil tensiometer installed at a precisely georeferenced position) and not obtained through spatial interpolation, with short-term forecasts, filtered and aggregated at the edge or in the cloud [6], to deliver timely, context-aware irrigation recommendations.

Within this context, agricultural decision support systems, and more specifically Irrigation Decision Support Systems (IDSS), have emerged as key technologies for enabling data-driven irrigation management [8]. These systems integrate real-time data on weather, soil moisture, and crop stress, possibly collected via IoT devices, to support optimized water use. Among the various soft computing techniques applied in IDSS, fuzzy logic has gained increasing traction due to its ability to handle uncertainty and imprecise data, as extensively reviewed by Patel et al. [9]. Fuzzy inference systems are particularly well-suited for translating heterogeneous sensor inputs into actionable irrigation strategies and for supporting rule-based, autonomous decision-making within decentralized IoT architectures.

However, many existing IDSS implementations, fuzzy-based or otherwise, still rely on static, physics-based models with limited integration of real-time data streams or predictive analytics [10]. A promising but underexplored direction is to combine the descriptive, rule-based reasoning of fuzzy logic with the adaptive capabilities of machine learning (ML) [11]. This hybrid approach could enable anticipatory irrigation decisions that better reflect evolving field conditions, reducing both water waste and crop stress.

In this work, we compare two fuzzy IDSS implementations: (i) a Mamdani-based inference engine with expert-defined rules [11], and (ii) a Takagi–Sugeno-based system introduced here, which enables automated learning from ultra-local field data. Both integrate predictive components from previously proposed machine learning models [12,13], deployed in the cloud. Field experiments show that both controllers reduce water use compared to traditional irrigation while keeping soil moisture within agronomic thresholds. The Mamdani-based system reduces the occurrence of critical dry days, providing slightly better soil moisture stability. In contrast, the ANFIS-based controller achieves greater water savings by adopting a more conservative irrigation strategy (i.e., applying less water). These results reveal a trade-off between maximizing water efficiency and practicing deficit irrigation (i.e., deliberately allowing for mild water stress), highlighting the practical relevance of both approaches depending on management priorities.

The remainder of this paper is organized as follows: Section 2 reviews related work on precision agriculture, with a focus on IDSSs and the application of fuzzy logic in water management; Section 3 presents the proposed fuzzy-logic-based systems and the comparison strategy; Section 4 describes the software–hardware platform, the study area, and the datasets used; Section 5 outlines the counterfactual simulation and bootstrap-based analysis used for assessing the performance of the systems; Section 6 discusses the comparative results and trade-offs; and Section 7 summarizes the findings and highlights future research directions.

## 2. Related Work

Advances in precision agriculture, such as remote sensing, variable-rate systems, autonomous machinery, IoT, and AI, are transforming farm management by enabling real-time monitoring, adaptive control, and data-driven decision-making [3,4,5,14]. These technologies have paved the way for decision support systems that integrate diverse data sources to provide practical, evidence-based recommendations for more efficient and sustainable agricultural practices [10,15].

In recent years, there has been growing attention among researchers, practitioners, and policymakers toward IDSS [16,17]. IDSS are designed to support farmers in making informed decisions about when and how much to irrigate, ensuring water is used precisely and efficiently. To achieve these goals, IDSS can integrate various data sources, such as weather forecasts, soil moisture data, and crop-specific water requirements, with advanced analytical and predictive methods [4]. In addition to improving water management, these systems significantly enhance farm profitability [18,19].

Rosillon et al. [20] propose a near real-time spatial interpolation method for air temperature and humidity, improving IDSS accuracy through kriging and reanalysis data [21]. Conde et al. [22] design an adaptive DSS that integrates human inputs to improve scheduling efficiency. In viticulture, Kang et al. [23] introduce a IDSS for regulated deficit irrigation in wine grapes, focusing on soil moisture monitoring. King et al. [24] present an IoT-based IDSS with a crop water stress index and neural networks for precision irrigation. Simionesei et al.  [8] presented an IDSS deployed in southern Portugal, which integrates data from local weather stations, 7-day weather forecasts, and the MOHID-Land soil water balance model [25].

Regarding the use of fuzzy logic in combination with IoT technology for IDSS, Patel et al. [9] present an autonomous irrigation device that processes multiple field inputs, including current weather conditions, air temperature, soil moisture, and water availability in a storage tank. The system employs a fuzzy inference engine with 81 manually defined rules to automate the opening and closing of a water valve, thereby optimizing irrigation schedules without human intervention. Similarly, Kokkonis et al. [26] propose an IoT-based irrigation device that performs local sensing and actuation through an embedded fuzzy inference system. Their system collects data from multiple soil moisture sensors, as well as air temperature and humidity sensors, each modeled using three fuzzy membership levels. The fuzzy logic algorithm, implemented directly on the microcontroller, determines the opening angle of a central servo valve to control irrigation. These approaches enable real-time, on-device decision-making without relying on constant connectivity, making them particularly suitable for deployment in remote or infrastructure-limited agricultural settings.

More recently, and in a manner closely aligned with our work, Benzaouia et al. [27] propose an intelligent IDSS that combines IoT-based environmental and soil sensing with a Mamdani-type fuzzy logic controller implemented directly on an ATmega2560 microcontroller. The system processes real-time inputs such as soil moisture, temperature, solar irradiance, and rainfall to dynamically determine optimal irrigation timing and duration. Meanwhile, the sensed and aggregated data are transmitted via LoRa communication to a cloud-based infrastructure for storage and visualization. The main objective is to improve water and energy efficiency in semi-arid agricultural settings. Field experiments conducted in a Moroccan apple orchard demonstrated that the fuzzy controller effectively reduced irrigation during periods of high evapotranspiration and adjusted watering durations in response to varying environmental conditions.

## 3. Fuzzy-Based Decision Support Systems

In this section, we present the two developed IDSSs based on fuzzy logic, which are later compared using real-world data. Fuzzy logic offers a powerful framework for reasoning under uncertainty, inspired by the way humans make decisions in the presence of imprecise or incomplete information [28]. Unlike classical binary logic, which imposes a strict true/false dichotomy, fuzzy logic allows variables to assume degrees of truth, enabling more nuanced and rule-based decision-making, therefore earning the motto of “computing with words” [29]. For the particular case of irrigation management, fuzzy logic offers a natural way to encode agronomic expertise into decision support tools. Its linguistic rule structures allow domain experts to define irrigation strategies in intuitive terms (e.g., “if soil is dry and high temperature is expected, then irrigate generously”), while the underlying inference engine translates these qualitative insights into quantitative actions.

The two IDSSs differ in their fuzzy inference methods: the first uses a Mamdani-type system known for its straightforward rule-based logic, while the second employs an Adaptive Neuro-Fuzzy Inference System (ANFIS), which combines fuzzy logic with neural networks. Despite these differences, both systems share the same four input variables and produce a single output variable. Specifically, the input variables are:

1. Last Avg Tensiometer: the current day’s average tensiometer reading (${\bar{S}}_{d}$), representing the most recent soil water tension, which is directly related to the soil moisture level;
2. Predicted Avg Tensiometer: the predicted average tensiometer reading for the following day (${\hat{S}}_{d+1}$), generated by ultra-local Long Short-Term Memory (LSTM) machine learning models trained on historical data [12,13];
3. Predicted Rain Amount: the predicted cumulative rainfall over the next three days (${\hat{R}}_{d,3}^{\mathrm{sum}}$), obtained from a weather forecast service;
4. Predicted Max Temperature: the maximum predicted air temperature over the next three days (${\hat{T}}_{d,3}^{\max}$), also obtained from a weather forecast service.

Regarding the output, this variable represents the recommended irrigation level. Depending on the irrigation system and the field layout (e.g., organized in rows or other configurations), it may carry different operational meanings. In this study, the output is defined as the duration of irrigation cycles relative to a reference vineyard row within a water sector, where each sector corresponds to a predefined and homogeneous area of the field as determined by agronomists.

The decision to employ a deep learning model architecture rather than classical physically based soil water-balance models (i.e., FAO-56 [30] or AquaCrop [31]) is driven by data compatibility and operational practicality. While physically based models provide mechanistic insights, they require precise soil parameters and crop coefficients, often uncertain at the local scale. In contrast, the adopted data-driven approach leverages tensiometric time series to model soil water tension directly, capturing complex, non-linear site-specific dynamics and facilitating deployment as new sensor data become available.

In the remainder of this section, we provide a detailed and formal description of the design and development of the two fuzzy systems analyzed and compared in this work.

### 3.1. Mamdani-Type Fuzzy IDSS

The first IDSS adopted in this study is the fuzzy inference model developed and validated for vineyard irrigation management in Northern Italy [11]. It addresses the critical need for sustainable water use and agronomic precision in a dynamic, weather-sensitive agricultural context.

**Figure 1:**
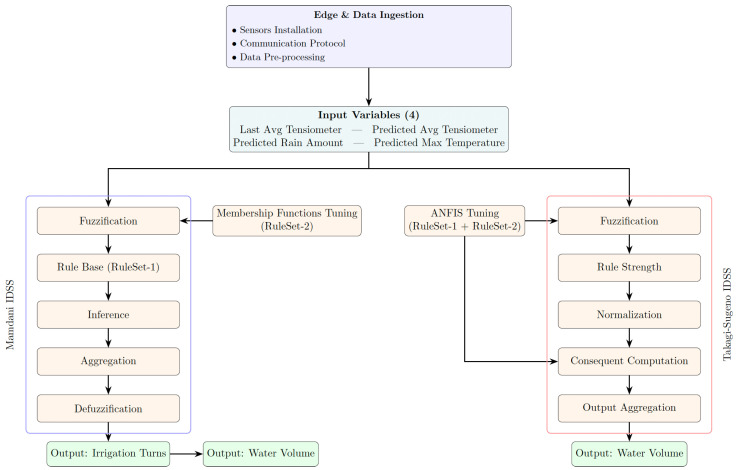
Workflow of the dual IDSS architecture, showing the Mamdani and ANFIS inference pipelines.

Formally, this IDSS is based on the classical Mamdani-type fuzzy system [32], which can be conceptually decomposed into five main components, schematically represented in the Mamdani branch of Figure 1 and briefly described below:

1. Fuzzification: it transforms crisp inputs $x_{i}$ into fuzzy values $\mu_{A_{i}}\left(x_{i}\right)$, where $x_{i}$ is the *i*-th input, and $\mu_{A_{i}}\left(x_{i}\right)$ is the membership function of the fuzzy set $A_{i}$.
2. Rule Base: it defines rules of the following form:
  $$R_{k}:\;\mathrm{IF}\;x_{1}\;\mathrm{is}\;A_{1}^{k}\;\wedge \;\ldots \;\wedge \;x_{n}\;\mathrm{is}\;A_{n}^{k}\;\mathrm{THEN}\;y\;\mathrm{is}\;B^{k},$$
  where *k* indexes the rules, $A_{i}^{k}$ are fuzzy sets for the inputs, and $B^{k}$ is a fuzzy set for the output.
3. Inference block: it computes the degree of activation for each rule using fuzzy logic operators:
  $$\mu_{B^{k}}\left(y\right)=\min\left(\mu_{A_{1}^{k}}\left(x_{1}\right),\mu_{A_{2}^{k}}\left(x_{2}\right),\ldots \right),$$
  for the AND operator (alternative operators like OR may use max).
4. Aggregation: it combines the fuzzy outputs from all rules:
  $$\mu_{B}\left(y\right)=\max_{k}\mu_{B^{k}}\left(y\right),$$
  where $\mu_{B}\left(y\right)$ is the aggregated membership function.
5. Defuzzification: it converts the aggregated fuzzy set $\mu_{B}\left(y\right)$ into a crisp output *y*. The method adopted in our case is the centroid:
  $$y=\frac{\int y\cdot \mu_{B}\left(y\right)\;dy}{\int \mu_{B}\left(y\right)\;dy}.$$

More specifically, each input variable is fuzzified into three linguistic terms (*Low*, *Medium*, and *High*), while the output variable is defined by four linguistic terms corresponding to the standard irrigation turns, namely *No irrigation*, *Half-turn*, *Single-turn*, and *Double-turn*. In operational terms, a *Single-turn* corresponds to a specific irrigation duration per vineyard row of the reference sector, which in our system represents the application of 650 L of water; the other irrigation turns are defined proportionally to this standard. For all variables, the linguistic terms at the extremes are modeled using trapezoidal membership functions, whereas the intermediate terms are modeled using triangular membership functions (see Table 1 for a summary of input variable domains and the fuzzy-set peaks used in the experiments).

Finally, the rule base of this IDSS comprises the same 21 original fuzzy rules described in [11], developed collaboratively with agronomists and irrigation managers to coherently represent various environmental scenarios and ensure comprehensive coverage of critical soil and meteorological conditions of this study area. In the following, this set of rules will be referred to as Ruleset-1. For illustrative purposes, one representative rule from Ruleset-1 is shown below:

$$\begin{array}{cc} R_{i}:\; & \mathrm{IF}\;\mathrm{Last}\;\mathrm{Avg}\;\mathrm{Tensiometer}\;\mathrm{is}\;\mathrm{High}\\ & \;\wedge \;\mathrm{Predicted}\;\mathrm{Avg}\;\mathrm{Tensiometer}\;\mathrm{is}\;\mathrm{High}\\ & \;\wedge \;\mathrm{Predicted}\;\mathrm{Rain}\;\mathrm{Amount}\;\mathrm{is}\;\mathrm{Low}\\ & \;\wedge \;\mathrm{Predicted}\;\mathrm{Max}\;\mathrm{Temperature}\;\mathrm{is}\;\mathrm{High}\\ & \mathrm{THEN}\;\mathrm{Irrigation}\;\mathrm{Suggested}\;\mathrm{is}\;\mathrm{Double}\;\mathrm{Turn} \end{array}$$

### 3.2. Takagi-Sugeno Fuzzy IDSS

The second IDSS proposed in this paper is based on the Adaptive Neuro-Fuzzy Inference System (ANFIS). Formally, this system employs first-order Sugeno-type rules [33] and follows the original five-layer architecture introduced in [34]. In essence, ANFIS combines the transparent, rule-based reasoning of fuzzy logic with the learning capabilities of artificial neural networks. Instead of manually defining the rules and the parameters (e.g., the shapes) of the linguistic terms, the system automatically learns them from the example data.

The same input and output variables defined at the beginning of this section are used here. However, ANFIS directly produces a crisp output, expressing the irrigation recommendation as a numerical value. In our case, this value corresponds to the number of liters of water to be applied per vineyard row.

More in detail, the first-order Sugeno rule base comprises rules of the following form:

$$R_{k}:\;\mathrm{IF}\;x_{1}\;\mathrm{is}\;A_{1}^{k}\;\wedge \;\ldots \;\wedge \;x_{n}\;\mathrm{is}\;A_{n}^{k}\;\mathrm{THEN}\;y_{k}=\sum_{i=1}^{n}p_{i}^{k}\;x_{i}\;+\;r^{k},$$

where each fuzzy set $A_{i}^{k}$ may take any differentiable shape (e.g., triangular, trapezoidal, Gaussian), and $p_{i}^{k}$ are linear coefficients optimized during training. The final output is produced by aggregating these local consequents via their normalized firing strengths.

The network comprises five layers, namely:

- Layer 1-Fuzzification: Each crisp input $x_{i}$ is mapped to a set of membership values $\mu_{A_{i}^{k}}\left(x_{i}\right)$ through parameterized membership functions. The shape parameters (e.g., centers, widths, slopes) are initialized heuristically and refined through learning.
- Layer 2-Rule Strength: For each rule *k*, the firing strength $w_{k}$ is computed as the t-norm (typically the product or minimum) of the antecedent membership degrees:
  $$w_{k}=\overset{n}{\underset{i=1}{⨂}}\mu_{A_{i}^{k}}\left(x_{i}\right).$$
- Layer 3-Normalization: Each rule’s firing strength is normalized across all rules:
  $${\bar{w}}_{k}=\frac{w_{k}}{\sum_{j=1}^{K}w_{j}},$$
  ensuring that $\sum_{k}{\bar{w}}_{k}=1$.
- Layer 4-Consequent Computation: The normalized strength ${\bar{w}}_{k}$ weights a local first-order polynomial function:
  $$f_{k}\left(x\right)=p_{1}^{k}x_{1}+p_{2}^{k}x_{2}+\cdots +p_{n}^{k}x_{n}+r^{k},$$
  where the coefficients $\left\{p_{i}^{k}\right\},r^{k}$ are learned jointly with the membership function parameters.
- Layer 5-Output Aggregation: The final crisp output is the weighted sum of the rule outputs:
  $$y=\sum_{k=1}^{K}{\bar{w}}_{k}f_{k}\left(x\right).$$

With ANFIS, training is performed end-to-end using gradient-based optimization (e.g., backpropagation to minimize the mean squared error), allowing the model to adjust the fuzzy partitions and the Sugeno consequents simultaneously. This joint optimization enables the inference system to capture complex, non-linear relationships among soil moisture levels and predictions, weather forecasts, and crop water demand, while maintaining a transparent rule-based structure.

### 3.3. IDSS Tuning Methodology

A key step in [11] for designing the Mamdani-type IDSS was the use of Bayesian optimization to fine-tune the membership function parameters (e.g., the shapes and centers of the linguistic terms). To this end, the authors created a validation dataset, hereafter referred to as Ruleset-2, based on expert feedback collected through structured surveys that simulated realistic irrigation scenarios. Specifically, in each survey, an expert was presented with a set of numerical values for the four input variables that represents a possible field status and was asked to indicate the corresponding number of irrigation turns to be applied. During the optimization process, each candidate fuzzy system was evaluated on Ruleset-2 by comparing its irrigation recommendations with those provided by the experts. The objective was to minimize the mean squared error (MSE), defined as:

(1) $$\mathrm{MSE}=\frac{1}{n}\sum_{i=1}^{n}{\left(y_{i}-{\hat{y}}_{i}\right)}^{2}$$

where $y_{i}$ is the expert’s recommendation and ${\hat{y}}_{i}$ is the corresponding output generated by the fuzzy system for the *i*-th validation sample.

In this study, to ensure an accurate and fair comparison between the two IDSSs, both were evaluated under identical conditions. For the ANFIS model, this required building a dedicated training dataset by combining Ruleset-1 and Ruleset-2. Since ANFIS operates on numerical input–output pairs rather than purely linguistic descriptors, each fuzzy rule from Ruleset-1 was systematically defuzzified into one or more crisp samples. Specifically, for every antecedent term, we selected between one and three numerical values corresponding to the points where its membership function reached its maximum before Bayesian tuning. When a rule antecedent did not constrain a particular variable (i.e., the linguistic category was unspecified), that variable was instantiated by generating one representative numerical value for each of its defined linguistic terms. This expansion converted the original fuzzy rule into multiple concrete training examples. The procedure follows the approach described in [35], where membership-function peaks are sampled to generate numerical training data from a fuzzy rule base. Finally, the dataset was augmented with the expert-derived samples from Ruleset-2.

Finally, to determine the optimal configuration of the ANFIS-based IDSS, a cross-validation procedure is employed to tune its hyperparameters. The search space includes the type of membership functions used to model the linguistic terms, the learning rate of the training algorithm, and the number of training epochs. In each fold, model performance is evaluated using the MSE defined in Equation (1). The average MSE across all validation folds is used to select the best hyperparameter combination. Once cross-validation is complete, the model is retrained on the entire dataset with the selected hyperparameters, thereby leveraging all available data to refine the fuzzy partitions and decision surfaces under optimal learning conditions.

Figure 1 provides a graphical overview of the entire workflow, illustrating both the Mamdani and ANFIS inference pipelines.

## 4. Experimental Setup

This section details the experimental setup used to evaluate the objectives of this study. We describe the software platform that manages data acquisition and analysis, followed by a description of the data collected from the field experiments.

### 4.1. The Data-Oriented Software Platform

**Figure 2:**
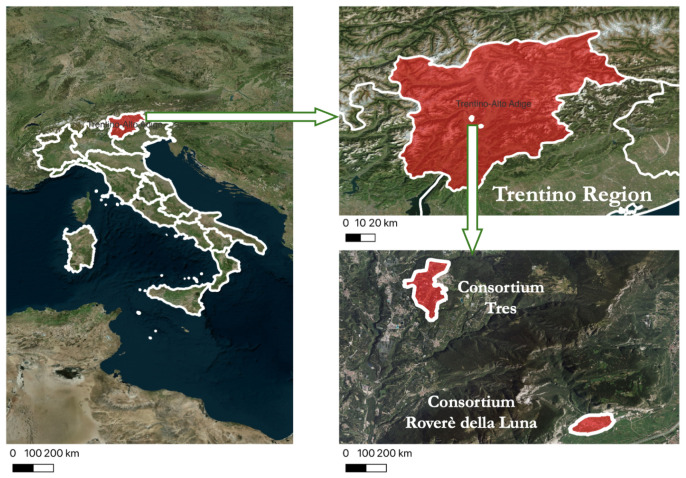
Geographic coverage of the IDSSs deployed in the Trentino region.

The comparative evaluation of the fuzzy IDSSs is conducted using the IrriTre platform [36]. Briefly, the latter is a cloud-based system that currently monitors over a thousand agricultural fields in the Trentino region of northern Italy. These fields are organized into irrigation consortia, each managed under a common water distribution infrastructure. Figure 2 illustrates two example consortia and their geographic locations in the region.

**Figure 3:**
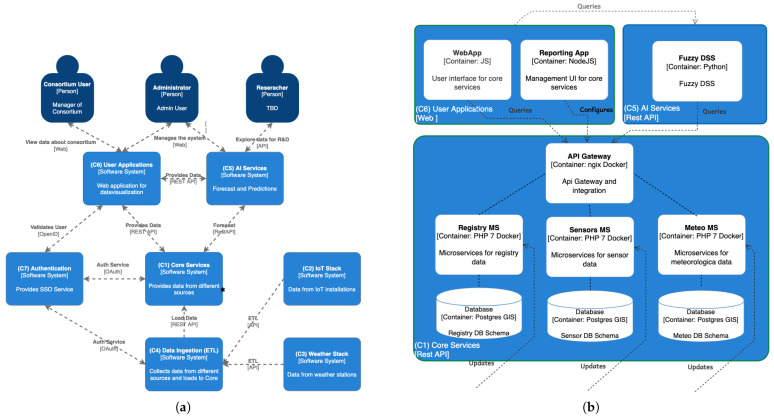
IrriTre platform architecture and component interactions. (**a**) Context diagram. (**b**) Container diagram for C1, C5 and C6.

The IrriTre software platform adopts a modular microservice architecture [37] for irrigation decision support, integrating sensor networks, AI-based analytics, and data management via several REST APIs [38]. It leverages open-source components that are widely adopted in industry to reduce costs, enhance flexibility, accelerate development, and avoid vendor lock-in. As illustrated in Figure 3a, it consists of seven software modules, briefly described below.

#### 4.1.1. Core Services (C1)

This group of services acts as the central hub for data management. They are composed of three distinct modules, namely Registry, Sensors, and Meteo, aggregating and serving data from heterogeneous sources, as illustrated in Figure 3b. The Registry service manages cadastral data, including both company registration (business profile) and geographic information, while the Sensors and Meteo microservices maintain records of IoT sensors and weather stations, respectively. To ensure maximum interoperability, all entities conform to the OGC SensorThings API standard [39].

#### 4.1.2. IoT Stack (C2)

This microservice forms the backbone of the platform’s sensor infrastructure, leveraging low-power and long-range wireless communication technologies. It currently supports over 200 sensors, including tensiometers, water flow meters, and pulse counters, which have been continuously collecting data since early 2023.

**Figure 4:**
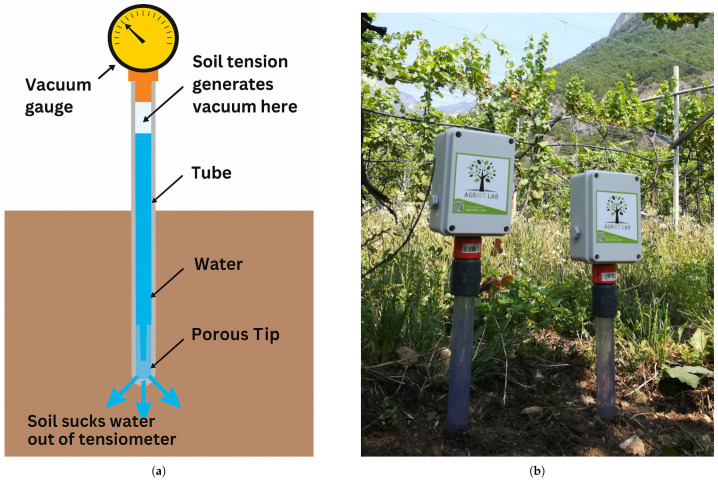
Tensiometers used in the IrriTre platform to measure soil moisture tension. (**a**) A schematic view of a tensiometer showing the main parts of the device, including the water-filled tube, porous ceramic cup, and vacuum gauge used to detect soil water tension. (**b**) Two tensiometers deployed at different soil depths (30 and 60 cm) in an agricultural plot.

Specifically regarding tensiometers, they are employed in IrriTre to measure soil water tension due to their accuracy, cost effectiveness, and low sensitivity to environmental variability [40,41]. As illustrated in Figure 4, a tensiometer consists of a water-filled tube, a porous ceramic cup, and a vacuum gauge to detect negative soil pressure. As the soil dries, water is drawn out of the ceramic cup, generating a negative pressure inside the tube that is proportional to the soil water potential.

Within IrriTre  each tensiometer installed in the soil measures soil water tension at two depths (30 and 60 cm) at 15-min intervals. All measurements are stored locally on a battery-powered microcontroller and transmitted via LoRaWAN to geographically distributed IoT gateways that are part of the IrriTre territorial sensor network.

#### 4.1.3. Weather Stack (C3)

**Figure 5:**
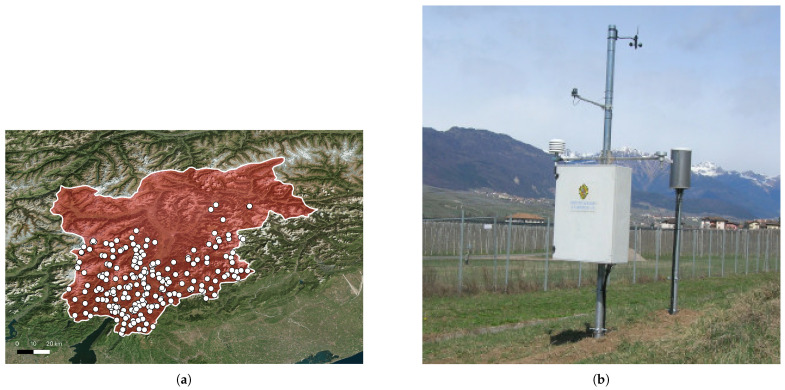
Weather stack. (**a**) Geographic distribution of the weather stations deployed in the Trentino sub-region (the southern part of the red-highlighted area in the figure; the northern part corresponds to the Alto Adige sub-region). White dots indicate the locations of individual weather stations. (**b**) An example of a deployed weather station, taken from [42].

This microservice is responsible for retrieving and storing weather data from a public network of over 250 meteorological stations geographically distributed across the Trentino region. As shown in Figure 5, this network, managed by the Municipality of Trento and the Fondazione Edmund Mach research institution [42], provides extensive regional coverage. Regarding the nature of collected data, weather variables are recorded hourly across the network and include standard environmental metrics: air temperature at 2 m above ground, relative humidity, wind speed and direction, precipitation, and solar radiation. These data are particularly valuable for agricultural decision-support services like frost alerts and regional forecasts.

#### 4.1.4. Data Ingestion (C4)

This microservice is responsible for collecting, transforming, and loading data from various sources, such as IoT sensor nodes and weather station networks, into the Core Services layer. This process is commonly referred to as ETL, from the acronym Extract-Transform-Load. By decoupling data acquisition from downstream processing and storage, the ETL microservice significantly enhances the reliability, scalability, and maintainability of the overall data pipeline [43]. This layer supports both batch and near-real-time data flows and includes mechanisms for data validation, normalization, and temporal alignment.

#### 4.1.5. AI Services (C5)

This group of services represents the intelligence layer of the IDSSs, as it provides forecasting capabilities and predictive analytics. As shown in Figure 3b, these services consume processed data from the Core Services and return model outputs and predictions, enabling seamless integration with other components of the platform.

The analytics layer hosts a range of machine learning and statistical models tailored to support irrigation-related decision-making, including soil water tension forecasting, water demand estimation (the output of the IDSSs), telemetry, and anomaly detection tools. These models operate on historical and real-time data ingested from sensors and meteorological sources. In particular, our IDSSs based on fuzzy logic make use of predictive estimations of soil water tension measured by tensiometers. These predictions are generated using machine and deep learning models previously published in [12,13] (see Section 3). The combination of data-driven prediction and rule-based reasoning enables the platform to provide timely and context-aware irrigation recommendations, even in the presence of partial or delayed sensor data.

#### 4.1.6. User Applications (C6)

**Figure 6:**
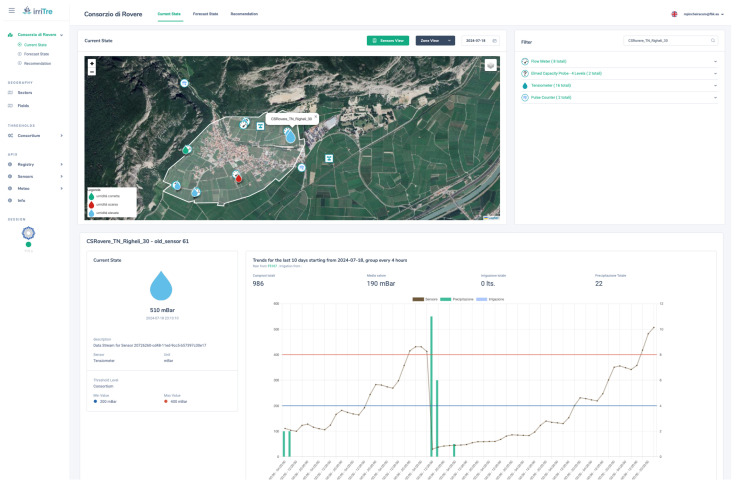
Web-based interface of the IrriTre platform for irrigation monitoring and control, showing a dynamic map with sensor locations and a dashboard summarizing real-time soil tension data from a selected tensiometer.

This component includes various user-facing applications for dashboarding, reporting, and monitoring. In particular, a web-based interface allows users to interact with the data and insights generated by the AI services and the Core Services. Currently, in IrriTre it is implemented as a responsive web application featuring comparison charts, interactive dashboards, and maps displaying the location of each sensor, as shown in Figure 6.

#### 4.1.7. Identity and Access Control (C7)

Three user roles are defined for the IrriTre platform, namely Consortium Users, Administrators, and Researchers. Each role interacts with a specific module through web interfaces or APIs, depending on its purpose (management, exploration, or data access). The Authentication service provides Single Sign-On (SSO) using OAuth and OpenID, using multiple identity providers.

### 4.2. Study Area and Dataset Description

For this study, we used a subset of the data available on the IrriTre platform. The selected geographic area includes vineyard regions, making it particularly relevant for evaluating irrigation practices in viticulture. The time frame from 1 May 2023 to 31 August 2023, was chosen as it coincides with the critical irrigation period of the vine-growing season. This phase is crucial, as soil moisture management during this period significantly influences vine health, yield, and grape (and consequently wine) quality.

Soil tension readings collected by tensiometers during this period ranged from approximately 15 to 650 mbar, reflecting a wide variety of field conditions. This range is significant, as typical irrigation thresholds for grapevines, defined by the local agronomists, lie between 200 mbar (indicating sufficient moisture) and 400 mbar (indicating dry conditions). It therefore enables an effective assessment of irrigation strategies, including those focused on water-stress management. Meteorological data relevant to this study were retrieved from available weather stations, while meteorological forecasts were obtained from OpenMeteo and stored in the IrriTre platform.

**Figure 7:**
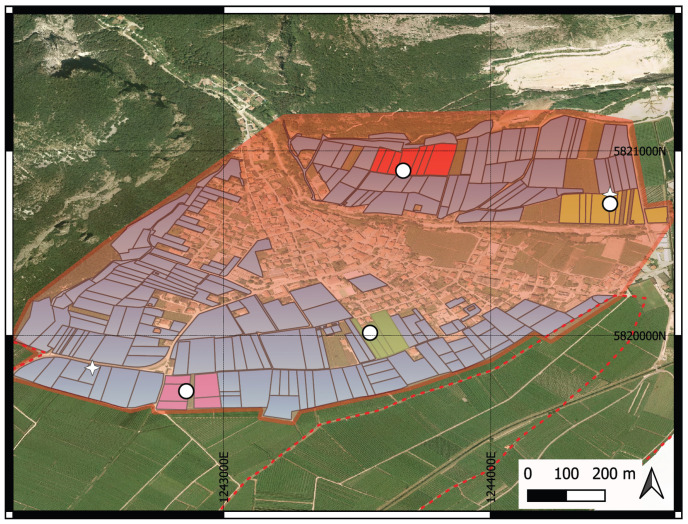
Geographic map of the study area. White dots represent the location of the sensors, while the stars represent the location of the weather stations.

The analysis focuses on four representative vineyard sectors, examining tensiometer data and associated sprinkler operations to evaluate irrigation performance across the fields. Figure 7 depicts the geographic map of the study area, showing the outline of the municipality (light red), the irrigation consortium (gradient light blue), the sectors under study (pastel colors), and the locations of the four tensiometers and two weather stations (circles and four-pointed stars, respectively).

## 5. Evaluation Framework

To assess the effectiveness and robustness of the two fuzzy IDSSs under realistic and uncertain field conditions, we adopt a two-part evaluation framework. First, in Section 5.1, a counterfactual simulation is used to estimate how each system would have performed under real historical conditions, enabling a dynamic, scenario-based comparison of irrigation outcomes. Then, in Section 5.2, a bootstrap-based statistical analysis quantifies the significance of observed differences by accounting for variability in environmental factors.

### 5.1. Counterfactual “What-If” Analysis

The counterfactual “What-If” simulation illustrated in Algorithm 1 is conducted to evaluate the behavioral performance of the two IDSSs in a dynamic and real-world context [44]. This methodology is aligned with the evaluation framework proposed in [11], where a similar iterative simulation was used to assess the Mamdani-type fuzzy IDSS. In that study, predictive models were trained on historical data to simulate tensiometer responses, and the system was evaluated by comparing its suggestions with expert decisions. Consistent with that approach, the present analysis considers multiple field sectors and leverages temporally grounded soil tension predictions to drive decisions.
**Algorithm 1** Daily Counterfactual Simulation and Irrigation Decision Loop  1:**Input:** Current average soil tension ${\bar{S}}_{d}$; weather data (factors) $F_{1},F_{2},\ldots ,F_{J}$  2:**Output:** Simulated soil tension ${\hat{S}}_{d+1}$; irrigation recommendation ${\hat{I}}_{d+1}$  3:**for** each day d=1 to *N* **do**  4:      **Step 1:** Simulate soil tension with the LSTM model assuming  5:                   no irrigation  6:           Predict ${\hat{S}}_{d+1}=f\left(F_{1},F_{2},\ldots ,F_{J}\right)$ assuming $I_{d+1}=0$  7:      **Step 2:** Evaluate irrigation recommendation with IDSS  8:           Construct state vector $x=({\bar{S}}_{d},{\hat{S}}_{d+1},{\hat{R}}_{d,3}^{tot},{\hat{T}}_{d,3}^{tot})$  9:           Compute ${\hat{I}}_{d+1}=\mathrm{IDSS}\left(x\right)$10:      **Step 3:** Evaluate irrigation condition and update soil tension11:      **if** ${\hat{I}}_{d+1}\neq 0$ **then**12:           Update ${\hat{S}}_{d+1}$ to account for ${\hat{I}}_{d+1}$13:      **else**14:           Proceed without irrigation; retain predicted ${\hat{S}}_{d+1}$15:      **end if**16:**end for**

In essence, the process simulates how soil moisture levels would have changed over time if the system’s recommendations had been followed, using the same historical environmental conditions. It operates through a daily simulation loop, where the IDSS is run retrospectively on past data to generate irrigation suggestions. The impact of these suggestions on soil moisture is then estimated, allowing the influence of the system on moisture dynamics to be evaluated over time.

The process is iterative and, at each simulation step, the model first forecasts the next day’s soil tension, assuming that no irrigation is performed. This counterfactual baseline serves as input to IDSS, which then generates its irrigation recommendation. If irrigation is advised, the simulated soil tension is updated to reflect the estimated hydrological response to the applied water volume. Otherwise, the simulation advances to the next day without modification. This process is repeated over the entire evaluation period, thereby emulating the operational deployment of the IDSS across a continuous time horizon.

The performance of the system under simulated deployment is assessed using three core metrics: (i) the total volume of irrigation water applied (expressed in liters per vineyard row); (ii) the number of critical dry days (defined as number of days on which soil water tension exceeded a predefined dryness threshold); and (iii) the average soil water tension level maintained during the simulation window (measured as the mean soil water tension in millibars). These three indicators jointly reflect an IDSS’s ability to optimize the trade-off between water efficiency and agronomic reliability, key criteria in sustainable irrigation management.

### 5.2. Bootstrap-Based Statistical Analysis

To assess the statistical robustness and significance of the IDSSs performance, a non-parametric bootstrap framework [45] is adopted. Bootstrap resampling is performed on the multivariate weather factor series ${\left\{F_{d,j}\right\}}_{d=1,\ldots ,N}^{j=1,\ldots ,J}$, with a stratified moving block approach applied to preserve temporal dependence [46], as depicted in Algorithm 2.
**Algorithm 2** Stratified Block Bootstrap Loop  1:**Input:** Weather factor series $\left\{F_{d,j}\right\}$ with d=1,…,N, j=1,…,J; block size *w*; number of temporal strata *K*; number of samples *B*  2:**Output:** Empirical distribution of IDSS performance values ${\left\{M^{\left(b\right)}\right\}}_{b=1}^{B}$  3:**for** each sample b=1 to *B* **do**  4:      **Step 1:** Generate stratified bootstrap sample  5:           Partition timeline into *K* strata of consecutive days  6:           Extract all overlapping blocks of length *w* from $\left\{F_{d,j}\right\}$  7:           Assign each block to its corresponding stratum *k*  8:      **for** each stratum k=1 to *K* **do**  9:           Draw $M_{k}$ block start indices ${\left\{s_{k,m}^{\left(b\right)}\right\}}_{m=1}^{M_{k}}$ from stratum *k* with10:           replacement11:      **end for**12:      Concatenate sampled blocks in temporal order into ${\left\{F_{d,j}^{\left(b\right)}\right\}}_{d=1}^{N}$13:      **Step 2:** Evaluate IDSS performance14:           Run counterfactual simulation (Algorithm 1) on $\left\{F_{d,j}^{\left(b\right)}\right\}$15:           Compute performance metric $M^{\left(b\right)}$16:**end for**

The method accounts for temporal dependencies by extracting overlapping blocks of fixed length *w*, grouped into *K* consecutive temporal strata. Within each stratum, blocks are sampled with replacement and concatenated in chronological order to form synthetic weather trajectories. The complete procedure generates *B* bootstrap samples and evaluates each through a simulation-based counterfactual loop (e.g., Algorithm 1).

Each resampled trajectory $\left\{F_{d,j}^{\left(b\right)}\right\}$ is fed into the same IDSS evaluation pipeline as the original data. Specifically, the IDSS processes each input configuration in sequence, producing irrigation decisions that recursively update the simulated soil moisture state. The resulting performance score for sample *b* is denoted $M^{\left(b\right)}$, and the collection ${\left\{M^{\left(b\right)}\right\}}_{b=1}^{B}$ forms an empirical distribution from which we derive confidence intervals based on percentiles (1−α), using the empirical α/2 and 1−α/2 quantiles.

Finally, to determine whether the performance difference between the two IDSSs is statistically significant, a bootstrap hypothesis test is performed by estimating the fraction of samples in which one variant outperforms the other [46]. The bootstrap *p*-value is computed as:

$$p=\frac{1}{B}\sum_{b=1}^{B}1\left\{M_{A}^{\left(b\right)}<M_{B}^{\left(b\right)}\right\},$$

where $M_{A}^{\left(b\right)}$ and $M_{B}^{\left(b\right)}$ denote the performance of configurations A and B on the *b*-th sample.

This non-parametric test accounts for temporal dependencies and avoids distributional assumptions.

## 6. Results and Discussion

The simulations and analyses were fully implemented in Python (version 3.11). For specific software libraries, Scikit-Fuzzy was used for Mamdani-based fuzzy logic modeling, while the S-ANFIS library in PyTorch (version 2.9.1) was employed for implementing the Takagi-Sugeno ANFIS model.

The optimal hyperparameter configuration of the ANFIS-based IDSS was identified by a grid search, in which each candidate was evaluated using a k-fold cross-validation procedure (with k=5). Before the ANFIS learning phase, all input and output variables were standardized to zero mean and unit variance. The grid search explored different types of membership function, learning rate values, and numbers of training epochs, as summarized in Table 2. Each candidate configuration was evaluated by averaging the mean squared error over the five folds.

The best performing configuration used the hybrid membership function shape (two sigmoidal flanks with a central Gaussian peak), a learning rate of $10^{-3}$, and 200 epochs. In this setting, the cross-validation process produced a standardized average MSE of 0.118 (see Equation (1)). These results demonstrate that hybrid fuzzy partitions better reflect the natural behavior of the input variables: the sigmoidal parts capture gradual changes that stabilize at the extremes, while the Gaussian peak provides precise focus around key central values. Using these hyperparameters, the model was finally re-trained on the full dataset to refine fuzzy partitions and decision surfaces.

**Figure 8:**
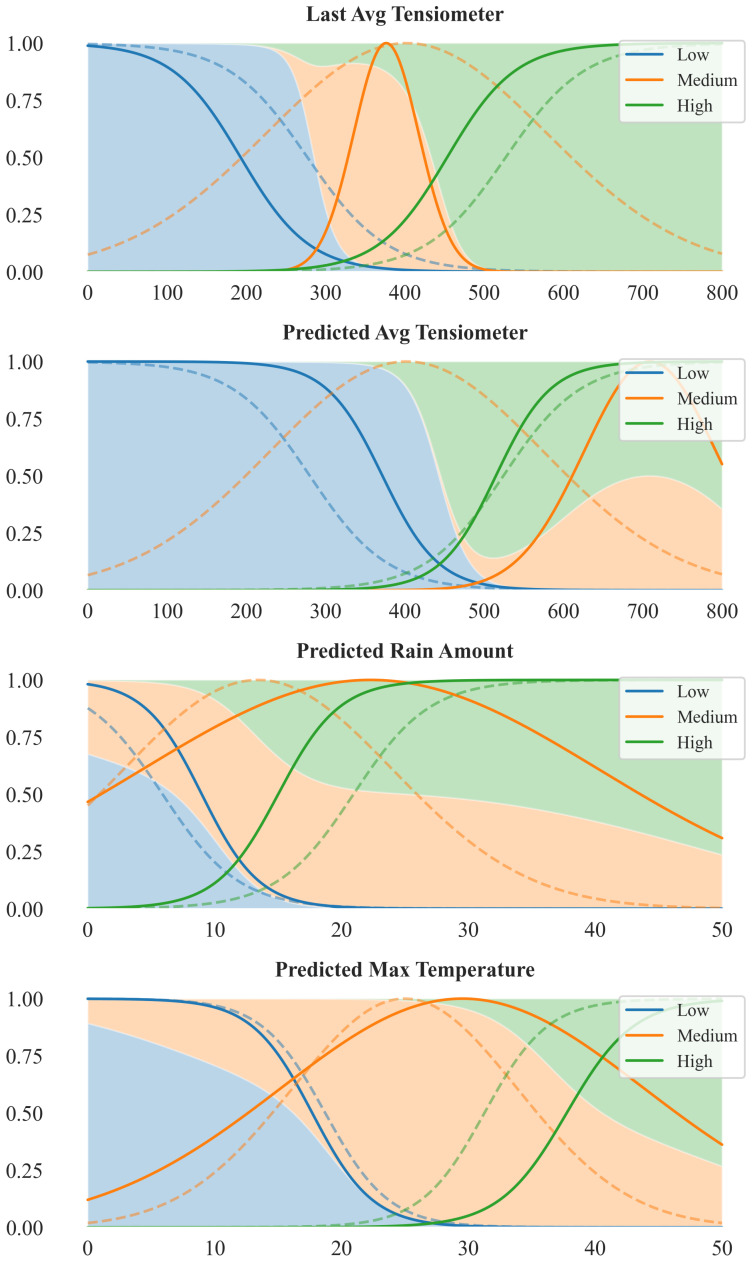
Evolution of the membership functions in the ANFIS-based IDSS. Dashed lines indicate the initial membership functions, while solid lines represent the functions after the training phase. The x-axis reports the physical variable (Last Avg Tensiometer [mbar], Predicted Avg Tensiometer [mbar], Predicted Rain Amount [mm], Predicted Max Temperature [°C]), while the y-axis indicates the Degree of Membership.

Figure 8 illustrates the changes from the initially defined membership functions (based on domain-expert knowledge and aligned with [11]) to those obtained after training, shown as dashed and solid lines, respectively.

After training, the membership functions of the *Last Avg Tensiometer* show a pronounced refinement: the *Low* term becomes sharply confined below approximately 250 millibars (mbar), and the *High* function rises rapidly just above 400 mbar, drastically reducing the transition zone. This indicates that the model has learned to strongly emphasize recent soil water tension measurements in identifying water stress thresholds. In contrast, the *Predicted Avg Tensiometer* variable maintains broader and smoother membership transitions. The three linguistic terms remain largely overlapping, with the *Medium* membership retaining a relatively flat and wide distribution, suggesting a more diffuse contribution of the forecasted moisture values to the model decision boundaries. For the *Predicted Rain Amount* variable, the membership functions contract around the moderate precipitation range (roughly 10–25 mm). Both *Low* and *High* memberships pull away from extremes, indicating a focus on central rainfall values as the most informative for the model’s output. Regarding *Predicted Max Temperature* variable, the optimized membership functions remain largely similar to their initial configuration. While minor sharpening occurs, particularly around the *Medium* region, the overall shape and boundaries of the curves are preserved, suggesting that the initial partitioning was already well aligned with the structure of the data in this dimension.

**Figure 9:**
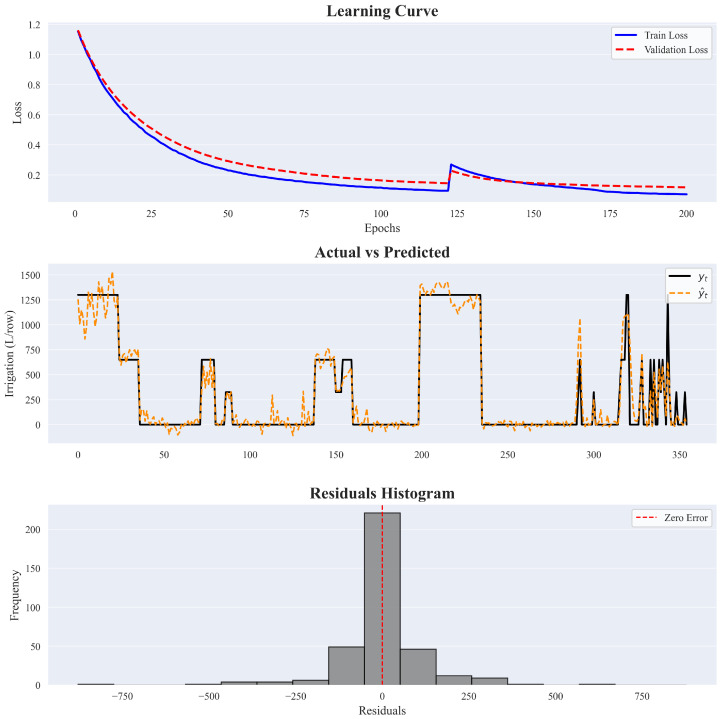
Training Dynamics and Performance Assessment of the ANFIS Model. Shown are the learning curves (**top**), actual vs predicted values (**middle**), and residuals histogram (**bottom**).

Figure 9 provides an overview of the model’s training process along with an evaluation of its adaptation accuracy. The first subplot illustrates the evolution of the training and validation errors, expressed as the mean standardized MSE across the five cross-validation folds. The learning curves reveal a generally decreasing trend, with both errors dropping steadily over time. However, a noticeable spike occurs around the 125th epoch, where both training and validation losses increase abruptly. This likely reflects a temporary instability during training, possibly due to an unfavorable update in the rule parameters or a temporary overfitting to certain folds. Despite this, the model quickly recovers, and both error curves resume a downward trajectory, ultimately reaching a stable minimum at the latest epochs. In particular, the training and validation curves remain consistently close throughout the process, suggesting that the model does not suffer from significant overfitting.

The second subplot shows the relationship between observed and predicted irrigation volumes after the ANFIS model was retrained on the full dataset, represented by solid black and dashed orange lines, respectively. The outputs shown are continuous values; in operational deployment, they are mapped to the nearest valid discrete irrigation volume. While this transformation is not depicted in Figure 9, it is a critical step for integration into the decision-making process. As shown in the plot, the model aligns well with all irrigation demands, for both low and high water volumes, indicating a strong fit to the data.

The third subplot shows the histogram of residuals, where each residual is defined as $r_{i}=y_{i}-{\hat{y}}_{i}$. The distribution is centered around zero, with a high concentration of small errors, indicating accurate model predictions in most cases. The symmetry of the residual distribution suggests that the model does not systematically overestimate or underestimate the suggested irrigation volumes, ensuring balanced predictions. Considering that a single irrigation turn corresponds to 650 L/row, the absolute prediction error exceeds this value only twice, once with a positive residual and once with a negative residual.

**Figure 10:**
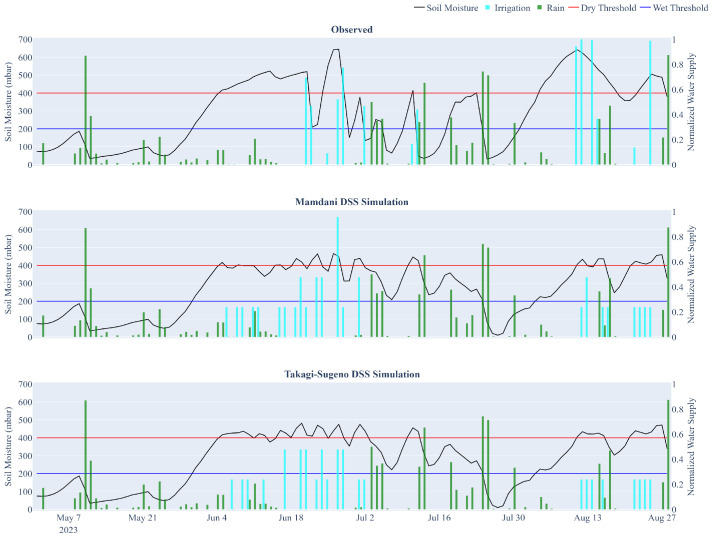
Comparison of actual and IDSS-simulated irrigation schedules for a specific water sector in the study area. The top subplot shows real tensiometer readings, rain, and irrigation events; the middle subplot presents simulations from the Mamdani-type IDSS; and the bottom subplot depicts simulations from the Takagi–Sugeno ANFIS IDSS. The red and blue horizontal lines represent the dry and wet thresholds defined by the agronomists.

Figure 10 illustrates an example comparison of irrigation scheduling for a single water sector in the study area during the 2023 season, contrasting the actual farmer-managed strategy with simulations produced by the Mamdani-type IDSS and the Takagi–Sugeno ANFIS IDSS (IDSS_M_ and IDSS_TS_, respectively).

Both IDSSs improve upon the observed irrigation schedule by keeping soil water tension more consistently within the target range shown in Figure 10 (blue and red lines). Values between these two lines represent the optimal moisture window for crop health. In the observed strategy (top subplot), soil tension frequently drifts outside this range, with several irrigation events occurring shortly before or after substantial rainfall (cyan and green bars, respectively). In contrast, the IDSS simulations distribute water more effectively over time, reduce overlaps with rainfall, and produce tensiometer readings that are generally more stable. Notably, IDSS_TS_ adopts a more conservative, water-saving approach compared to IDSS_M_, tending to delay irrigation events and applying slightly lower water volumes, while accepting marginally higher tensiometer readings (i.e., drier soil) in exchange for reducing irrigation volumes.

The same analysis was conducted for four water sectors in the study area, with the results summarized in Table 3. On average, the observed strategy applied 1,663 L of water per row, compared to 6,175 L for IDSS_M_ and 5,444 L for IDSS_TS_, corresponding to water savings of 51.25% and 57.03%, respectively.

As shown in Table 3, despite the reduction in water use, both IDSSs maintained effective moisture control. The number of days exceeding the critical dryness threshold decreased, on average, from 27.8 under observed management to 15 with IDSS_M_ and 23 with IDSS_TS_. As expected, the average tensiometer readings increased moderately, from 237.3 mbar to 244 mbar for IDSS_M_ and 250.3 mbar for IDSS_TS_, confirming more efficient water use while keeping soil tension within agronomic thresholds.

Across all sectors, both decision support strategies consistently outperformed the observed strategy in terms of water use efficiency, with IDSS_TS_ systematically using less water than the Mamdani-based variant. Interestingly, sector-level patterns reveal the adaptive behaviors of the controllers. In Sector 2, the second most water-intensive sector under the observed management (11,486 L of water used), both IDSS variants halved water volume (5,200 and 4,875 L for IDSS_M_ and IDSS_TS_, respectively), critical dry days dropped significantly and the average tensiometer readings remained near 250 mbar in all cases. Instead, in Sector 4, the sector with the highest water stress, IDSS_TS_ applied 6,825 L (versus 8,450 L for IDSS_M_) but incurred 33 critical days, exceeding even the observed strategy. In general, these results confirm that both IDSSs produce robust and adaptive irrigation strategies: IDSS_M_ achieves stronger dryness control per unit of water saved, while IDSS_TS_ maximizes volume reduction, with an increase in stress days.

To assess the statistical robustness of the differences observed between the decision systems, a stratified block bootstrap procedure described in Section 5.2 was implemented. The analysis was based on soil moisture tensiometer data from Sector 4 (i.e., the one depicted in Figure 10), which was selected because it is considered the most stable and responsive to field variations among all monitored sectors. A total of 1,000 simulations were generated through stratified resampling of the original 2023 weather series. Each trajectory consisted of 123 days, divided into K=4 temporal strata corresponding to the four summer months, and segmented into overlapping blocks of w=7 days to capture short-term autocorrelation in weather patterns. The resulting ⌊123/7⌋=17 blocks (plus four remaining days) were sampled with replacement within each stratum and concatenated chronologically to preserve realistic temporal dependencies.

**Figure 11:**
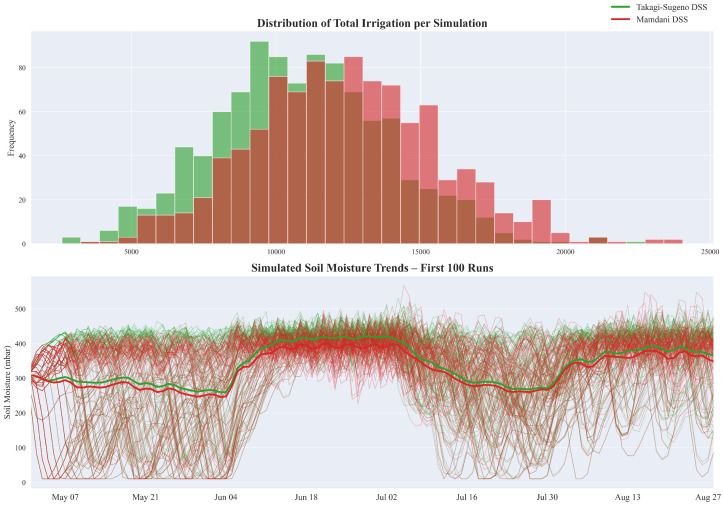
Distribution of total irrigation and simulated soil water tension trends for bootstrap simulation. The red and green colors represent the two IDSSs, while the intermediate shade in the histogram results from their overlap.

Figure 11 displays the bootstrap distributions of key performance metrics. The top plot shows the total irrigation volume per bootstrap sample, revealing that IDSS_TS_ consistently applies less water than IDSS_M_. The two distributions are clearly separated, with IDSS_TS_ shifted to the left, indicating a more conservative irrigation behavior under the same weather uncertainty. The plot below shows the corresponding distribution of the average soil water tension level. In this case, IDSS_M_ tends to maintain lower tensiometric values than IDSS_TS_, which implies slightly wetter soil conditions. Although both controllers operate within agronomic thresholds, overlap in the distributions seems to be more pronounced here than for irrigation.

To statistically validate these differences, one-sided pairwise bootstrap tests were performed at a level of significance of 5%. Table 4 reports the corresponding means, standard deviations, 95% confidence intervals, and *p*-values for the hypothesis of differences in average values. A statistically significant result supports the alternative hypothesis that the IDSSs perform differently in that respective metric.

In terms of total irrigation volume, IDSS_TS_ applied substantially less water than IDSS_M_, with a mean of 10,801.7 L/row compared to 12,349 L/row. The 95% confidence intervals for the two systems do not completely overlap, and the associated one-sided *p*-value of 0.001 indicates a statistically significant advantage for IDSS_TS_ in minimizing water use. However, IDSS_M_ shows better performance in reducing both the number of critical days (27.6 versus 46) and the average soil tension (327.8 mbar versus 342.1 mbar), indicating more favorable soil moisture conditions compared to IDSS_TS_. In both cases, the differences were supported by highly significant *p*-values.

These findings highlight a clear trade-off between the two decision support systems. IDSS_TS_ shows superior performance in minimizing water consumption, while IDSS_M_ is more effective in maintaining favorable soil moisture conditions. The bootstrap-based analysis provides robust support for these conclusions by accounting for variability in weather-driven environmental conditions and confirming the operational distinctions between the two approaches.

## 7. Conclusions

This work compared two fuzzy-logic–based IDSSs for vineyard management, namely a Mamdani-type controller with expert-defined rules and a Takagi–Sugeno ANFIS trained on ultra-local data. They are consistently defined and tested within a unified evaluation framework that combines counterfactual simulation and a stratified block bootstrap. Both systems integrate soil water tension sensing and short-term forecasts, and when employed in real settings, they reduce water use while keeping soil moisture within agronomic bounds.

Across four water sectors of a real agricultural consortium, the analysis reveals a clear trade-off: the ANFIS-based IDSS achieved greater water savings, whereas the Mamdani system better mitigated plant stress, reducing critical dry days and maintaining a lower average tensiometer value (e.g., wetter soil). These complementary strengths were observed consistently in both the campaign-level comparison and the bootstrap-based statistical analysis.

From a numerical standpoint, the ANFIS strategy reduced irrigation by around 57% on average compared to the baseline, a result supported by robust statistical analysis. In practical terms, if a slight increase in stress days is acceptable (for instance, it is a realistic trade-off for a resilient crop such as grapevine), the ANFIS controller is preferable. Conversely, for crops where water stress is less tolerable, the Mamdani controller may represent a safer choice.

From an operational perspective, this translates into a practical selection rule: when water scarcity or pumping costs are the dominant constraints, the ANFIS controller offers the greatest benefits; when minimizing crop stress and ensuring tighter moisture control is the highest priority, the Mamdani controller holds the advantage. Since both approaches are interpretable and already integrated with the IrriTre cloud platform, they can be deployed under different water management policies, or even combined in policy-driven ensembles.

Building upon the site-specific models developed in this study, future research efforts should also concentrate on enhancing their generalizability and transferability across diverse viticultural environments. Since the current models were trained exclusively under specific local conditions (e.g., local soil properties, climate, and irrigation management), a critical research direction is the data augmentation of the existing training set. This expansion would involve systematically collecting and integrating local data from a broader range of geographic areas and management regimes. The ultimate objective is model generalization: the development of robust, generalized models capable of providing reliable predictions and effective decision support across a significantly broader range of contexts, thereby minimizing the need for extensive site-specific recalibration.

## Figures and Tables

**Table 1:** Domains and membership-function peaks for input variables.

Fuzzy Variable	Domain	Peaks (Low, Medium, High)
Last Avg Tensiometer [mbar]	[0, 800]	{136, 300, 545}
Predicted Avg Tensiometer [mbar]	[0, 800]	{0, 300, 711}
Predicted Rain Amount [mm]	[0, 50]	{3, 15, 40}
Predicted Max Temperature [°C]	[0, 50]	{18, 20, 50}

**Table 2:** Grid search domains for ANFIS hyperparameters; the best-performing configuration found is highlighted in bold.

Hyperparameter	Domain
Membership function	Gaussian; Sigmoid; Generalized Bell; **Hybrid (Sigmoid + Gaussian)**
Learning rate	$\left\{10^{-3},\;10^{-4},\;10^{-5},\;10^{-6}\right\}$
Training epochs	{100,200,500,1000}

**Table 3:** Comparison of irrigation performance (in terms of water volume, critical days, and average tensiometer values) across the four sectors of the study area.

Sector	Water Volume per Row (L/row)			Critical Days			Avg Tensiometer (mbar)		
	Observed	IDSS_M_	IDSS_TS_	Observed	IDSS_M_	IDSS_TS_	Observed	IDSS_M_	IDSS_TS_
1	4,479	1,300	975	9	3	3	174	184	185
2	11,486	5,200	4,875	29	9	18	245	253	260
3	10,931	9,750	9,100	44	33	38	291	270	276
4	23,757	8,450	6,825	29	15	33	239	269	280
Total	50,653	24,700	21,775	111	60	92	949	976	1,001
Average	12,663	6,175	5,444	27.8	15.0	23.0	237.3	244.0	250.3

**Table 4:** Comparison of IDSSs based on the bootstrap analysis. All tests are one-sided: $H_{0}:\mu_{TS}\geq \mu_{M}$ vs. $H_{1}:\mu_{TS}<\mu_{M}$; an asterisk indicates that the test was performed in the reverse direction.

Metric	IDSS_M_: Mean (SD) [95% CI]	IDSS_TS_: Mean (SD) [95% CI]	*p*-Value ($H_{0}:\mu_{1}<\mu_{2}$)
Total irrigation (L/row)	12,349.0 (3,195.1) [6,500, 18,850]	10,801.7 (2,988.9) [5,200, 16,900]	0.0010
Critical days	27.6 (6.1) [16, 40]	46.0 (10.0) [27, 67]	<0.0001 *
Avg Tensiometer (mbar)	327.8 (25.6) [271.9, 371.3]	342.1 (28.5) [279.5, 391.0]	<0.0001 *

## Data Availability

The datasets presented in this article are not readily available because they are generated within a commercial project. Requests to access the datasets should be directed to fantonelli@fbk.eu.
